# Matching of Nitrogen Enhancement and Photosynthetic Efficiency by Arbuscular Mycorrhiza in Maize (*Zea mays* L.) in Relation to Organic Fertilizer Type

**DOI:** 10.3390/plants11030369

**Published:** 2022-01-29

**Authors:** Suravoot Yooyongwech, Waraporn Threeprom, Rujira Tisarum, Thapanee Samphumphuang, Daonapa Chungloo, Suriyan Cha-um

**Affiliations:** 1 School of Interdisciplinary Studies (Kanchanaburi Campus), Mahidol University, Kanchanaburi 71150, Thailand; wthreeprom@yahoo.com; 2 National Center for Genetic Engineering and Biotechnology (BIOTEC), National Science and Technology Development Agency (NSTDA), Pathum Thani 12120, Thailand; rujira.tis@biotec.or.th (R.T.); thapanee@biotec.or.th (T.S.); daonapa.chu@biotec.or.th (D.C.); suriyanc@biotec.or.th (S.C.-u.)

**Keywords:** arbuscular mycorrhizal fungi, nitrogen content, photosynthetic efficiency, organic fertilizer, maize

## Abstract

In the present study, *Funneliformis mosseae* (FM), *Claroideoglomus etunicatum* (CE), and *Acaulospora foveata* (AF) were inoculated to hybrid maize (*Zea mays* L. cv. CP888^®^). Upregulation of nitrogen levels were dependent on the type of mycorrhiza (AMF). Photosynthetic efficiency (F_v_/F_m_) and water content in FM- and AF-inoculated plants were elevated, resulting in promotion of leaf area and shoot biomass. N content in the shoot and root tissues of the FM-inoculated plants increased by 21% and 30% over the control. A positive correlation between biochemical, physiological, and morphological parameters using Pearson’s coefficient was demonstrated. A decline in lipid peroxidation was noticed in the FM-inoculated plants. In addition, we investigated the potential of N fertilizer application in combination with FM inoculation in maize plants. The FM-inoculated plants with organic O_LT, a chicken manure fertilizer, increased N content in the host shoots by 73% over the control, leading to improved F_v_/F_m_ as a physiological adaptation strategy. The FM and the O_LT on the regulation of the N enhancement and photosynthetic efficiency of the hybrid maize should further be validated in field trials in different environments for sustainability.

## 1. Introduction

Nitrogen, phosphorus, and potassium are major nutrients that regulate plant growth and development as well as plant phenology in crop species [1,2]. Nitrogen in different forms of fertilizer (i.e., ammonium (NH_4_^+^) and nitrate (NO_3_^−^)) has widely been applied in soil for plant cultivation and production [3,4]. In general, a 26% increase in production in major crops is linked to nitrogen fertilizer [5]. Excessive use of N fertilizer application and improper application methods result in reduced N use efficiency and increased N leaching, especially in maize cultivation [6]. It is thus imperative to optimize N implementation techniques that prevent N loss in the maize root zone [7]. The response of N fertilizer can regulate N status in plants and is associated with a prolonged green in maize leaves that serves the agricultural strategy of optimizing N use efficiency [8]. There are many low-input technologies for improving N availability in soil, such as N-fixing legumes (green manure and legume crop rotation), AMF-root colonized plants, and soil microbe nitrogen transformation, leading to the retention of crop productivity [9].

As regards microbe symbionts, AMF have a role as a biofertilizer and serve to support a plant’ quality advantage in agroecosystems [10]. At most, AMF symbiosis is able to improve a plant’s physiological traits (i.e., photosynthetic efficiency, plant biomass, and yield [11,12,13]), and AMF also influence N uptake into the host plant [14,15]. Nonetheless, the N content in the host plant depends on the AMF taxa as observed with *Acaulospora scrobiculata, Glomus etunicatum, G. mosseae,* and *G. versiforme* symbiosis in walnut [16]. AMF taxa have specific functional regulation of N availability in soil, and plant performance varies with the type of AMF taxa used [17]. In addition, the N uptake facilitated by AMF mycelium, such as *Rhizophagus irregularis*, was not enough to maintain a sufficient N status in the host plants [18,19]. It is, thus, imperative to investigate various AMF taxa and N sources with the aim of maximizing the efficiency of an AMF-specific genus and N source.

Maize is one of the most important cereal crops, and it is widely cultivated in several regions of the world: USA (38.5%), China (20.7%), Brazil (9.1%), European Union (5.8%), and Argentina (4.0%) [20]. It serves as raw materials of food, feed, and fuel (bioethanol) for the world’s population, and its production is predicted to increase to 9735 million tons by the year 2050 [21]. Increased productivity of maize using hybrid genotypes (single cross- and double cross-breeding programs) is one of the most conventional strategies to fill the gap [22,23]. At present, hybrid CP888^®^ maize, a dominate genotype widely distributed at >80% of the total area of maize cultivation in Thailand, Laos, Cambodia, and Vietnam [24,25], was selected as the candidate cultivar.

Recently, the reduced use of chemical-based agriculture cultivation techniques has become a trend in sustainable environmental crop production including maize. Divergent AMF that reflect an efficiency in N enhancement with photosynthetic regulation, pointing to a sufficient N status in the plant host for its development, together with alternative N sources was purposed. Therefore, this study investigated the potential of three AMF taxa—*Funneliformis mossea*, *Claroideoglomus etunicatum*, and *Acaulospora foveata*—and two organic N sources that matched in regard to N improvement and photosynthetic efficiency involving physiological fluctuations and growth characteristics in the hybrid CP888^®^ maize.

## 2. Results

### 2.1. AMF Colonization, N Content, and Plant Adaptation

**Figure 1:**
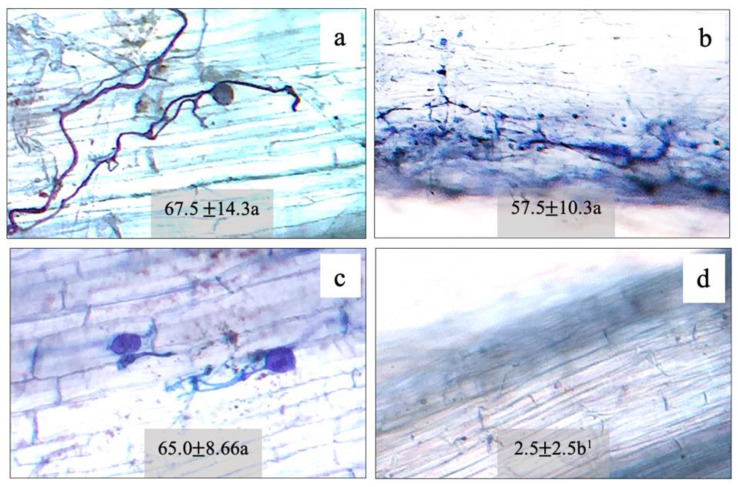
Morphological characters and root colonization of arbuscular mycorrhizal fungi: (**a**) *Funneliformis mosseae* (FM); (**b**) *Acaulospora foveata* (AF); (**c**) *Claroideoglomus etunicatum* (CE); (**d**) control (Con) in the hybrid maize host. ^1^ Mean ± SE. The different letters indicate significant data at *p* ≤ 0.01 using Duncan’s new multiple range test.

**Figure 2:**
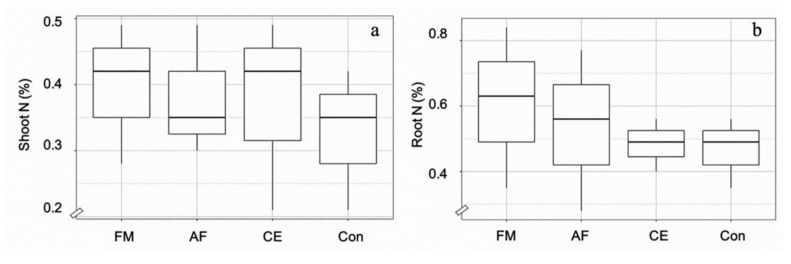
Box plots showing N content in the shoots (**a**) and roots (**b**) of the hybrid maize inoculated with *Funneliformis mosseae* (FM), *Acaulospora foveata* (AF), and *Claroideoglomus etunicatum* (CE) and the uninoculated control (Con). Data sorted by means.

Root colonization by *F. mosseae* (FA), *A. foveata* (AF), and *C. etunicatum* (CE) was observed to be 67.5%, 57.5%, and 65.0%, respectively, compared to the control (2.5%) (Figure 1). The hyphae and vesicle forms of FM, AF, and CE in the root tissues are given in Figure 1. The N content in the shoot and the root tissues of maize plants was regulated by all three types of AMF. In the shoots of plants inoculated with FM, AF, and CE, N content increased significantly by 21%, 21%, and 14% over the control, respectively; whereas, in the root tissues, it was enhanced by 30%, 15%, and 10%, respectively (Figure 2). N regulation was thus observed to be AMF-specific, especially in the roots.

**Figure 3:**
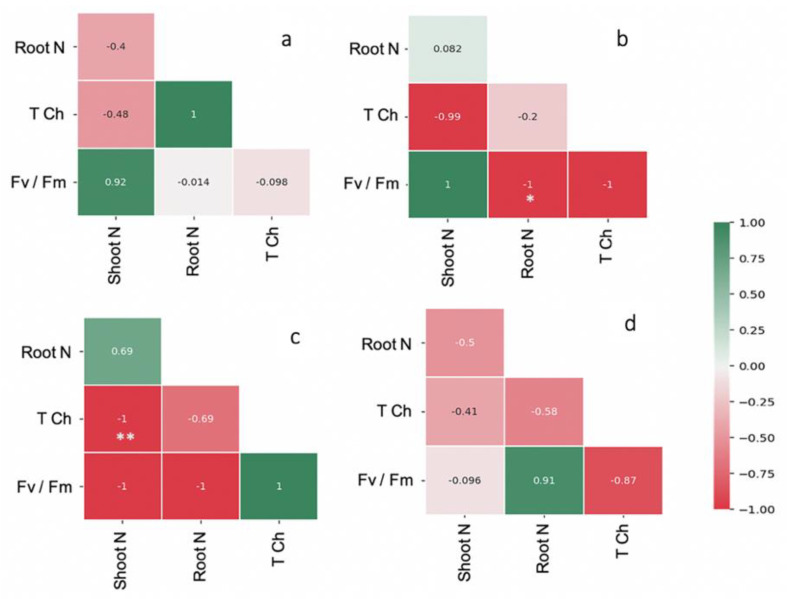
Pearson correlation coefficient matrices depicting relationship between N content and total chlorophyll content, photosynthetic efficiency (F_v_/F_m_) of the hybrid inoculated with (**a**) *Funneliformis mosseae* (FM), (**b**) *Acaulospora foveata* (AF), and (**c**) *Claroideoglomus etunicatum* (CE) and the (**d**) uninoculated control (Con). Scale from a −1 to 1 coefficient. Data were significant at ** *p* ≤ 0.01 and * *p* ≤ 0.05 using Duncan’s new multiple range test.

**Figure 4:**
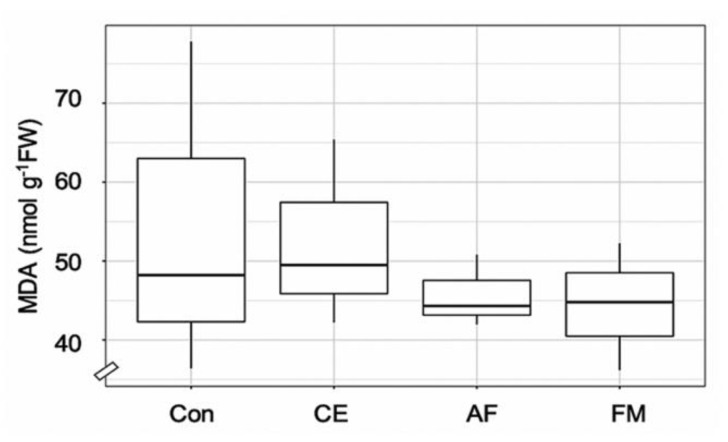
Box plot showing malondialdehyde (MDA) content in the shoots of the hybrid maize inoculated with *Claroideoglomus etunicatum* (CE), *Acaulospora foveata* (AF), and *Funneliformis mosseae* (FM) and the uninoculated control (Con). Data sorted by means.

The leaf area and shoot and root weights of FM-inoculated maize were higher than the control by 1.5-, 1.6-, and 1.3-fold, respectively (Table 1). The shoot and root WC and TCh content were highest in the maize plants inoculated with FM (i.e., 14.27 mg g^−1^, 62.42% and 52.74%; Table 1). The F_v_/F_m,_ maximum photosynthetic efficiency was regulated by AMF inoculation over the control (Table 1). Strong correlation coefficients were observed in FM-inoculated maize plants for shoot N and F_v_/F_m_ (*r* = 0.92) and root N and TCh (*r* = 1). Similarly, a strong correlation was observed in AF-inoculated maize plants between the shoot N and F_v_/F_m_ (*r* = 1), but a negative correlation was found in root N content and TCh. In the case of CE-inoculated plants, a negative correlation was observed between F_v_/F_m_ and TCh and N content in both shoots and roots (Figure 3). After the period of AMF symbiosis, the malondialdehyde (MDA) content in the shoots of FM-, AF-, and CE-inoculated plants was 17.97%, 15.59%, and 3.26% lower than in the control, respectively, indicating the protective effects of AMF under the environmental conditions (Figure 4). FM inoculation was the most effective in reducing the MDA content in maize plants (Figure 4).

**Figure 5:**
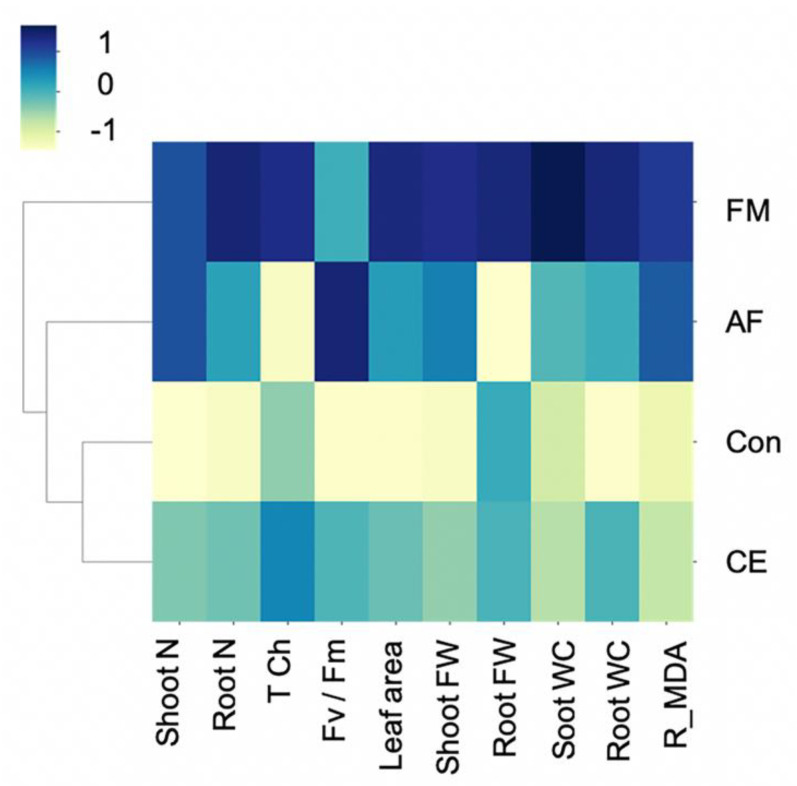
Heat map clustering (as Z-score values) of shoot and root N content, fresh weight, water content, total chlorophyll content, photosynthetic efficiency, and reversed MDA content of the hybrid maize inoculated with *Funneliformis mosseae* (FM), *Claroideoglomus etunicatum* (CE), and *Acaulospora foveata* (AF) and the uninoculated control (Con).

The clustering of an integration of the studied parameters was mapped to confirm the AMF-plant characteristics. Hierarchical clustering separated the plants’ physiological, biochemical, and growth parameters into three groups: (1) control (uninoculated) and CE-inoculated plants, (2) AF-inoculated plants, and (3) FM-inoculated plants. The greatest upregulation of growth characteristics was observed in the CP888^®^ maize inoculated with the FM-type AMF (Figure 5). In contrast, the characteristics of the CE-inoculated plants closely resembled those of the uninoculated control plants.

### 2.2. AMF Modulated N Improvement in Maize Plants

**Figure 6:**
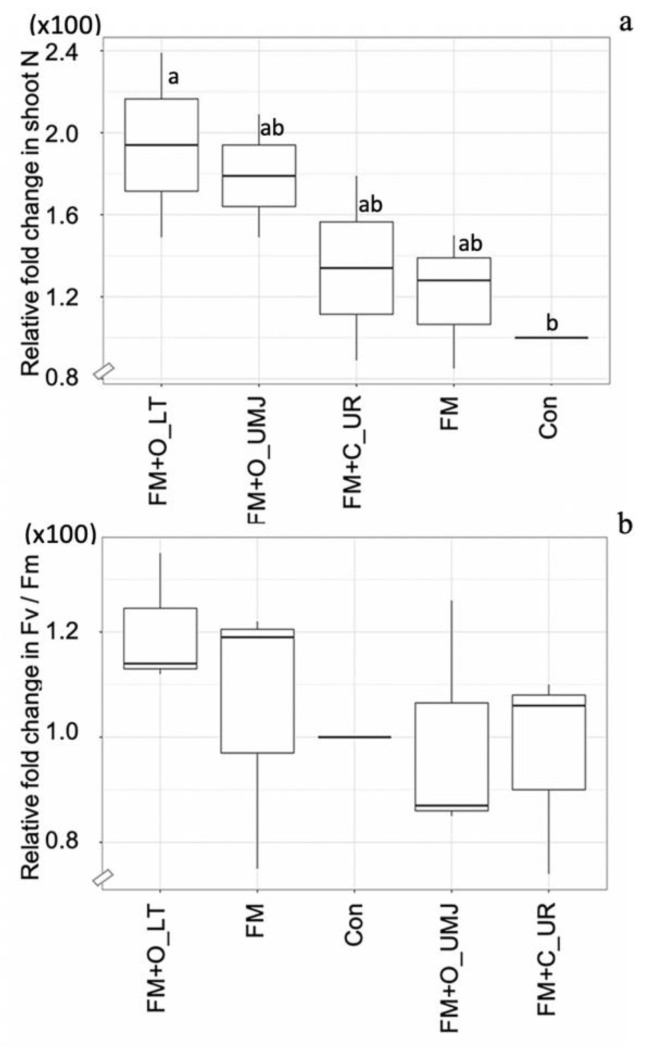
Box plots representing relative changes in N content (**a**) and photosynthetic efficiency (F_v_/F_m_) (**b**) in the shoots of the hybrid maize plant inoculated with *Funneliformis mosseae* (FM) with/without organic fertilizers (i.e., O_LT and O_UMJ), chemical fertilizer (i.e., C_UR), and the uninoculated control (Con). Data sorting by means. Different letters indicate significant differences at *p* ≤ 0.05 applying Duncan’s new multiple range test.

Based on the results of Experiment-I, FM was selected for Experiment-II. The relative N content in the shoots of the FM-inoculated plants with supplementation of the two organic fertilizers, O_LT and O_UMJ, increased by 73.57% and 58.57% and decreased slightly (1.42%) with supplementation of the chemical fertilizer (i.e., C_UR) compared to the control. However, the relative photosynthetic efficiency was remarkable in the FM-inoculated plants supplemented with organic fertilizer (O_LT) (Figure 6).

## 3. Discussion

Changes in the maize N response were assessed using different AMF (i.e., FM, AF, and CE) inoculation and two types of organic fertilizers (i.e., O_LT and O_UMJ) fertilizers. The N content in maize plants with/upon AMF inoculation might be related to the ability of AMF extraradical mycelium to modulate N_2_ uptake into intra-mycelium and, ultimately, to the host plant [14]. Variations in N uptake by different AMF species have also been observed in the roots of walnut, where the highest nitrogen uptake was mediated by inoculation of *Glomus etunicatum*, or CE, (15.86 g kg^−1^ DW), followed by *G. mosseae*, or FM, (9.78 g kg^−1^ DW) and *Acaulospora scrobiculata* (8.27 g kg^−1^ DW) [16]. Likewise, AMF-specific variations in N uptake were reported in mulberry by Shi et al. [26]. These variations might be due to the intraspecific diversity in the fungal N transporters and the plant host [27] as observed with AMF–maize symbiosis in the present study.

The grouping of the maize performance based on the intergraded framework of the plant characteristics revealed the differences in three AMF-inoculated maize. The greater performance of FM and AF with the host plant was corroborated by the investigation of Shi et al. [26]. In mulberry, *F. mosseae* and *A. scrobiculata* also depicted a positive response on growth improvement and development quality in total leaf N, photosynthetic pigment, and transpiration. On the other hand, shoot N and photosynthetic efficiency displayed strong positive correlations in both FM- and AF-inoculated plants. However, the strong positive correlation of root N with chlorophyll content was found only in the FM-inoculated plants. Wang et al. [28] suggested that FM inoculation was effective for improving N content in plants, including their roots, and chlorophyll content in *Gleditsia triacanthos* tree. As regards nitrogen improvement, the large amount of N flux in the AMF–host plants was reflected in the high carbon turnover to AMF intra-mycelium in the symbiosis [29]. In potato, with an increase in the N dose, a positive correlation between chlorophyll content and plant/tuber yield was observed, but the chlorophyll content and F_v_/F_m_ were negatively correlated [30]. Based on the obtained results, it is assumed that N regulation in the FM symbiosis takes precedence with high efficiency into root tissue compartments and a positive correlation in chlorophyll development compared to the AF and CE types.

MDA is a known indicator of cellular damage and is dependent on its cellular accumulation and dynamic regulation [31]. Reduction in MDA levels after AMF inoculation indicated involvement in the mitigation of plant membrane damage [32] through proteins and nucleic acids modification and interference with the photosystem and membrane fluidity, therefore leading to increased host plant development [31].

The increased N content of the plants supplemented with FM and two organic fertilizers plus the FM inoculation (Figure 6) was supported by a previous study reporting that the substantial ^15^N uptake was up to 31% into extraradical *Glomus* hyphae from a decomposed organic patch, implying and recognizing that AMF and organic material perform a role in the global N pool and are involved in N cycling [33]. It is possible that the increased N uptake in the FM-treated maize with the two organic fertilizers was more effective than the chemical fertilizer. However, the relative F_v_/F_m_ in the maize plants only showed the capability of the O_LT organic fertilizer, a chicken manure type. The reason for such an observation is not known; however, it is related to rich amino acid content in the chicken manure type (10.16–17.31% per dry weight), while cattle manure contained 7.61–10.89% amino acid content [34]. AMF (*Glomus hoi* and *G. intraradices*) degrade various types of organic N residues, proteins, and peptides into amino acid and translocate them from the extraradical mycelium to host plant [35]. In addition, an increase in soil enzyme activity highlight the influence of bio-organic fertilizer [36] related to AMF [37]. Moreover, organic plant manure was indicated to have higher induced soil enzyme activity rather than animal manure [38], while the enzyme activity in the organic chicken manure (i.e., O_LT) may be higher than in the plant plus cattle compost (i.e., O_UMJ), resulting in released N in plant–AMF symbiosis, (Figure 6). It was assumed that the supply of several mineralized N forms by O_LT organic sources was through the regulation of FM hyphae, leading to the efficiency of the soil enzyme activity, which was due both to improved N content and the photosynthetic efficiency in the FM plus O_LT plant.

Indeed, the altered N content and photosynthetic regulation were most distinguished in the results of the FM and the O_LT influences on the maize compared with previous reports as shown in Table 2. In this study, the FM may provide the utmost relevance to the O_LT fertilizer in terms of the plant–soil–microbe system, reflecting maize development over the long term, at least in Thailand’s climate. This may be supported by the balanced management of organic N fertilizer, which is positively correlated with the microorganism population and soil enzyme activity, resulting in improved plant yield, including soil–bio/physical properties over the long-term for sustainability [36,39].

## 4. Materials and Methods

### 4.1. Plant Material and Treatments

Seeds of maize (*Zea mays* L.; hybrid genotype CP888^®^, Charoen Pokphand Produce, Bangkok, Thailand) are commonly cultivated on sloping land in the north of Thailand [46]. Three seeds per pot were geminated and grown in sterilized soil. The soil (0.18% N, 0.12% P, 0.13% K, and 11.19% organic matter with pH 5.58 and an electro conductivity of 1.08 dS m^−1^) was sterilized using an autoclave at 121 °C for 15 min. For sterilization, it was mixed together with sand (1:2, *w*/*w*) poured into 12 inch plastic pots (2 kg substrate in each pot) with arbuscular mycorrhizal fungi inoculation. Two-hundred and fifty spores of mycorrhizal fungi, namely, (1) *Funneliformis mosseae* (T.H. Nicolson & Gerd.) C. Walker & A. Schüßler, formerly known as *Glomus mosseae*, hereafter FM; (2) *Claroideoglomus etunicatum* (W.N. Becker & Gerd.) C. Walker & A. Schüßler, formerly known as *G. etunicatum*, hereafter CE; (3) *Acaulospora foveata* (Trappe & Janos), hereafter AF, were inoculated before planting as Experiment I. After two months (V4 stage of corn vegetative growth), the root samples were collected for AMF colonization, and leaf N content, lipid peroxidation, photosynthetic abilities, and growth measurements were determined.

In Experiment II, a high potential AMF taxon from Experiment I was selected to evaluate symbiosis in combination with fertilizer utilization in terms of N content. Maize seedlings were germinated and grown in similar way as in Experiment I and inoculated with the best AMF type in combination with three N enriched fertilizers. The 1st treatment was organic fertilizer based on a chicken manure (4.30% N) (O_LT; Donk Bua^®^), and the 2nd treatment was organic fertilizer based on a plant plus cattle compost (5.08% N) (O_UMJ). The major nutrients and both the manures are given in Table S1. A positive check control treatment was included and indicated as chemical fertilizer (C_UR) composed of 2.43 g urea (46-0-0). Alternatively, the organic fertilizers, O_LT and O_UMJ, were autoclaved before the application. These organic fertilizers were applied at 26.06 and 22.06 g pot^−1^, according to recommendation by the Department of Agriculture, Thailand (218.75-kg N per hectare). The N content and photosynthetic efficiency in their leaves were evaluated and calculated relative to the change in control after two months (i.e., V4 stage).

### 4.2. AMF Colonization Analysis

AMF colonization was detected by mycorrhizal symbiosis in the roots of the hosted maize plants as per Yooyongwech et al. [13]. In brief, one centimeter of maize roots was collected and kept in 60% ethanol. Added to the root samples was 10% KOH (Sigma, Marlborough, MA, USA) and then boiled at 95 °C for 30 min. Next, root pieces were then cleaned and strained with 0.05% trypan blue (Sigma, Marlborough, MA, USA) for 15 min. The number of arbuscules, vesicles, and hyphae of the mycorrhizal fungi were practically processed and observed under a light microscope, and the percentage of the colonization was calculated.

### 4.3. Total N Determination

The N content in the leaf tissues was analyzed by the Kjeldahl method [47]. Five-hundred milligrams of shoot and root tissues were weighed and transferred into digestion tubes. Then, 20 mL of 36% sulfuric acid (Merck, NJ, USA) and 2 tablets of Kjeltab (Buchi, UK) were added to the tubes. The digestion was subsequently performed by heating at 420 °C for 50 min. After cooling the sample, 90 mL of deionized water and 80 mL of 32% NaOH (Sigma, Marlborough, MA, USA) were added. The distillation was then processed for 4 min. The ammonia released by water stream distillation was trapped in 80 mL of 2% H_3_BO_3_ (Sigma, Marlborough, MA, USA). It was then titrated against 0.05 mol L^−1^ H_2_SO_4_ until the color changed from green to violet. The % N was calculated using the equation:

N (%) = 1.4007 × C × (V − V_B_)/W,

where 1.4007—conversion factor, milliequivalent weight of N;

C—concentration of standard H_2_SO_4_ solution (mol L^−1^);

V—consumption of standard H_2_SO_4_ for sample (mL);

V_B_—consumption of standard;

H_2_SO_4_ for blank sample (mL);

W—initial sample weight (g).

### 4.4. Determination of Chlorophyll Content, Photosynthetic Efficiency, and Growth Character

Total chlorophyll content (TCh) in the second fully expanded leaf was analyzed as photosynthetic pigments. In brief, a hundred milligrams of leaf samples were weighed and transferred to test tubes. Then, 5 mL of 95% acetone was added (Sigma, Marlborough, MA, USA), homogenized, and kept in darkness at 4 °C for 48 h. The supernatant was collected, and the absorbance was recorded at 645, 662, and 470 nm, and the pigment content was calculated using the method of Shabala et al. [48].

A sensitive indicator of the photosynthetic performance in plants, a maximum photosynthetic efficiency (F_v_/F_m_), [49] were measured according to Maxwell and Johnson [50]. In brief, leaf clips were practically conducted, and dark adapted for 30 min on the leaf surface of the fully expand leaves (2–3rd upper leaf from shoot tip), and then the F_v_/F_m_ were recorded.

Shoot and root weights were measured, and the percentage of water content (WC) was calculated in shoot and root tissues according to the formula: % water content = (FW − DW)/FW × 100, where FW—fresh weight, and DW—dry weight [13]. In addition, the second to third leaves were selected to estimate the leaf area.

### 4.5. Malondialdehyde Analysis

A lipid peroxidation assay was determined by measuring the MDA content in the leaf tissues following the method given by Zhou et al. [51]. In brief, 0.5 g of shoot powder mixed with 3 mL of 50 mM potassium phosphate buffer (pH 7.8) were centrifuged at 12,000× *g* for 20 min. One milliliter of supernatant was collected and then added with 3 mL of 0.5% thiobarbituric acid (Sigma, Marlborough, MA, USA) and incubated at 95 °C in water bath for 60 min. After cooling, the absorbance was determined at 532 and 600 nm, and the MDA content was calculated using an extinction value of 155 mM^−1^ cm^−1^ and adapted to per gram fresh weight.

### 4.6. Experimental Layout and Statistical Analysis

In the experiment, the box plots were median, interquartile range at 25–75% with standard deviation, and the data values were sequenced by mean. One-way analysis of variance (ANOVA) was applied to test significant differences in different treatments using *n* = 3, except colonization (*n* = 6). Data were analyzed by post hoc Duncan’s new multiple range test. The Pearson correlation coefficient matrix was determined as a linear correlation between chlorophyll content and the photosynthetic efficiency related to the response of the N content. In addition, the linkages in N content, physiology, growth, and a reversed MDA were analyzed by hierarchical clustering. The statistical analyses were performed in Jamovi ver.1.6.18.0, an open-source software based on R (https://www.jamovi.org, accessed on 17 October 2021).

## 5. Conclusions

In this study, inoculation of maize plants with the AMF *F. mosseae* and *A. foveata* improved the maize’s characteristics. The *F. mosseae*-treated maize plants showed a positive correlation of root N content with chlorophyll content and shoot N with F_v_/F_m_. In addition, the *F. mosseae*-treated plants exhibited the lowest lipid peroxidation and played a vital role in N assimilation upon application with the organic fertilizers (i.e., O_UMJ and O_LT). However, the photosynthetic efficiency in the maize plants was sharply affected by *F. mosseae* plus the O_LT (chicken manure type). Moreover, the maize treated with *C. etunicatum* had a weaker development response and clustered closely with the untreated maize. Nevertheless, the ability of the AMF taxon *F. mosseae* in boosting plant N content and that of the organic fertilizer O_LT to regulate N implies an important role for the maize photosynthetic regulation for long-term sustainability.

## Figures and Tables

**Table 1:** Mean of total chlorophyll content (TCh), photosynthetic efficiency (F_v_/F_m_), leaf area, shoot and root fresh weight (FW), and their water content (WC) in shoots and roots of the hybrid maize inoculated with *Funneliformis mosseae* (FM), *Acaulospora foveata* (AF), and *Claroideoglomus etunicatum* (CE) and the uninoculated control (Con).

Treatment	TCh (mg g^−1^ FW)	F_v_/F_m_	Leaf Area (cm^2^)	Shoot FW (g plant^−1^)	Root FW (g plant^−1^)	Shoot WC (%)	Root WC (%)
Con	11.52 ± 2.05	0.61 ± 0.05	37.57 ± 8.12b	1.30 ± 0.39b	1.40 ± 0.14ab	43.73 ± 3.24b	42.17 ± 4.02b
FM	14.27 ± 2.25	0.65 ± 0.09	55.62 ± 18.18a	2.08 ± 0.59a	1.76 ± 0.30a	62.42 ± 4.24a	52.74 ± 11.31a
AF	10.01 ± 0.82	0.68 ± 0.02	49.19 ± 11.71ab	1.88 ± 0.28ab	0.87 ± 0.09b	45.76 ± 0.39b	48.30 ± 12.22ab
CE	13.01 ± 1.78	0.64 ± 0.09	45.98 ± 5.69ab	1.59 ± 0.24ab	1.37 ± 0.17ab	44.25 ± 7.33b	47.95 ± 3.36ab
*ANOVA*	ns	ns	*	*	**	**	*

Different letters in a column represent significant differences among treatments at *p* ≤ 0.05 applying Duncan’s new multiple range test. ns, *, and ** represent non-significant, significant at *p* ≤ 0.05, and significant at *p* ≤ 0.01, respectively.

**Table 2:** Study approaches with/without plant nitrogen (N) and photosynthetic regulation (PR), displaying AMF’s and/or fertilizer’s different influences compared to the current study.

Study Approach with/without, Involving N and PR	Priority Effective AMF	Fertilizer Consideration	Plant Host	Reference
N and PR	*Funneliformis mosseae*	Type comparing (Plant/animal manure organic and chemical fertilizer)	Maize (*Zea mays* L.)	This study
N and PR	*Glomus etunicatum*, *Diversispora spurca*	ns ^1^	Walnuts (*Juglans regia* L.)	[16]
N and PR (Under drought)	*Rhizophagus irregularis*	ns (Chemical dose)	Maize (*Zea mays* L.)	[19]
N and PR	*Funneliformis mosseae*, *Acaulospora scrobiculata*	ns	Mulberry (*Morus alba* L.)	[26]
N without PR	*Mix of Rhizophagus irregularis and Funneliformis mosseae*	Type comparing (Plant residue and mineral fertilizer)	Durum wheat (*Triticum durum* Desf.)	[15]
N without PR	*Rhizophagus irregularis*	ns (Chemical dose)	Poplar *Populus×canadensis*)	[40]
N without PR	*Rhizophagus irregularis*	ns	Poplar (*Populus×canadensis*)	[41]
N without PR	*Rhizophagus aggregatum* (plus Rhizobium)	ns (Plant organic fertilizer test)	Maize (*Zea mays* L.)	[42]
N without PR	*Rhizophagus irregularis*	ns	Legumes (*Medicago spp*.)	[43]
Without N, PR (Dry matter, yield)	*Glomus aggregatum*	ns (Chemical dose)	Maize (*Zea mays* L.)	[44]
Without N, PR (Growth parameters, phosphorus uptake)	*Glomus* spp.	ns	Maize (*Zea mays* L.)	[45]

^1^ ns—Fertilizer types were not considered in study.

## Data Availability

Not applicable.

## Supplementary Materials

The following are available online at https://www.mdpi.com/article/10.3390/plants11030369/s1, Table S1: Major nutrients in the organic fertilizers.
