# Supplementary material for: Matching of Nitrogen Enhancement and Photosynthetic Efficiency by Arbuscular Mycorrhiza in Maize (Zea mays L.) in Relation to Organic Fertilizer Type
Source: Plants (Basel). 2022 Jan 29;11(3):369. doi: 10.3390/plants11030369 (PMC8838784; doi:10.3390/plants11030369)
Supplement: Supplementary file 1 [file plants-11-00369-s001.zip › plants-1549743-supplementary.pdf]

Supplementary

Table S1. Major nutrients in the organic fertilizers

Nutrition (%)	Organic fertilizers, O_LT	Organic fertilizers, O_UMJ
N	4.30	5.08
P	3.80	2.49
K	3.70	0.58
Ca	4.4	2.31
Mg	0.7	1.25
